# P-1899. Improvement in Provider Experiences from Baseline to Month 12 with Integrating Cabotegravir Long-Acting (CAB LA) for PrEP into Care in an Implementation Science Trial (PILLAR)

**DOI:** 10.1093/ofid/ofae631.2060

**Published:** 2025-01-29

**Authors:** Taimur H Khan, Jonathan White, Linda Mercado, Bo Li, Katherine L Nelson, Lisa Petty, Michael Acquadro, Nicola Barnes, William Lenderking, Neelima Jain, Heidi Swygard, Todd McKeon, Annemiek de Ruiter, Maggie Czarnogorski, Nanlesta Pilgrim

**Affiliations:** Fenway Health / The Fenway Institute, Boston, Massachusetts; Peter Shalit MD & Associates, Seattle, Washington; Valley AIDS Council, Harlingen, Texas; GSK, Collegeville, Pennsylvania; ViiV Healthcare, Durham, North Carolina; ViiV Healthcare, Durham, North Carolina; Evidera, Bethesda, Maryland; Evidera, Bethesda, Maryland; Evidera Inc., Bethesda, Maryland; GlaxoSmithKline, Collegeville, Pennsylvania; ViiV Healthcare, Durham, North Carolina; ViiV Healthcare, Durham, North Carolina; ViiV Healthcare., London, England, United Kingdom; ViiV Healthcare, Durham, North Carolina; ViiV Healthcare, Durham, North Carolina

## Abstract

**Background:**

Real world clinical experience with CAB LA, the first long-acting injectable medication for HIV prevention, might be helpful to alleviate provider implementation concerns. We evaluate changes in healthcare providers (HCPs) CAB LA implementation outcomes over 12 months.

**Figure:**
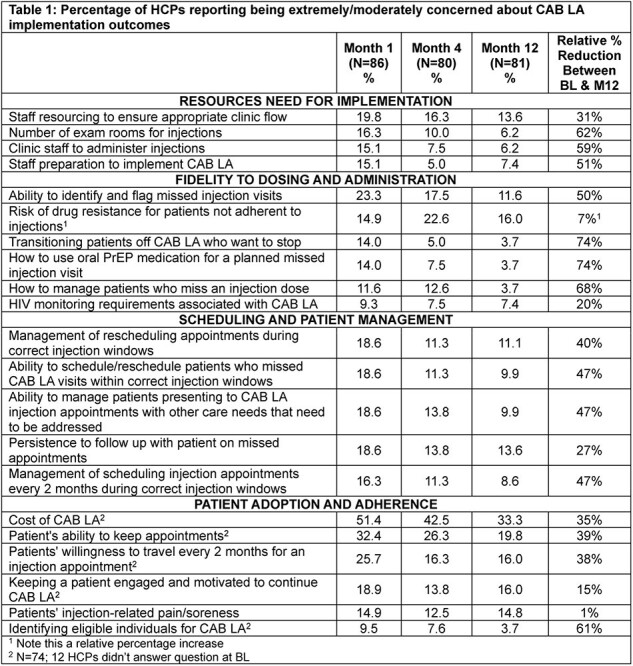

**Methods:**

PILLAR (ClinicalTrials.gov # NCT05374525) is a Phase IV randomized controlled trial conducted at 17 sites in the US. Sites were randomized 2:1 to dynamic (standard + enhanced support) and routine implementation (standard support). HCPs providing PrEP services were enrolled and completed surveys at month (M) 1, M4, and M12. Change across six CAB LA domains was assessed: 1) acceptability (4 items); 2) feasibility (4 items); 3) resources needed to implement (4 items); 4) fidelity to dosing and administration (6 items); 5) scheduling and patient management (5 items); and 6) patient adoption and adherence (6 items).

**Figure:**
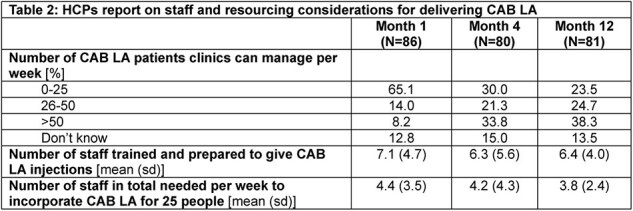

**Results:**

86 HCPs enrolled between April-October 2022 completed M1 surveys; 80 and 81 HCPs completed M4 and M12 surveys, respectively. HCPs reported high levels of acceptability and feasibility of CAB LA at M1 (mean scale scores≥4.4), M4 (mean scale scores≥4.1), and M12 (mean scale scores≥4.3) across both arms; change from BL to M12 between arms was not significant.

Across timepoints, HCPs reported reduced concerns in implementing CAB LA except risk of drug resistance due to non-adherence (Table 1). Concerns related to resources needed to implement CAB LA reduced by an average of 51% between M1 and M12. Staff needed per week to implement CAB LA reduced (M1: 4.4 vs. M12: 3.8) while the number of CAB LA patients clinics could manage per week increased (M1: >25=22% vs. M12: >25=63%) (Table 2). Concerns about scheduling and managing patients, fidelity to dosing and administration, and patients’ adoption and adherence reduced by an average of 42%, 46% and 32%, respectively, between M1 and M12. Drug resistance concerns rose by 7%.

A higher proportion of HCPs in the dynamic arm reported less concerns about resourcing and fidelity to dosing and administration than HCPs in the routine arm.

**Conclusion:**

HCPs confidence delivering CAB LA improved as early as M4 and continued through M12. Use of available implementation tools (e.g., guidance documents) support CAB LA implementation.

**Disclosures:**

Taimur H. Khan, MD MPH, ViiV Healthcare/GSK: Grant/Research Support Linda Mercado, PA-C, Gilead: Advisor/Consultant|Gilead: Grant/Research Support|Gilead: Honoraria|Gilead: Institution-samples; lunches for staff; travel support|Janssen: Institution-samples, lunches for staff|ViiV Healthcare: Advisor/Consultant|ViiV Healthcare: Grant/Research Support|ViiV Healthcare: Honoraria|ViiV Healthcare: Institution-samples; lunches for staff; travel support Bo Li, PhD, GSK: Employee Katherine L. Nelson, PhD, MPH, GSK: Stocks/Bonds (Public Company)|ViiV Healthcare: Employee Lisa Petty, MT(ASCP), GSK: Stocks/Bonds (Public Company)|ViiV Healthcare: Employee Michael Acquadro, PhD, GSK: Advisor/Consultant|ViiV Healthcare: Advisor/Consultant Nicola Barnes, MA, GSK: Advisor/Consultant|ViiV Healthcare: Advisor/Consultant William Lenderking, PhD, Gilead: Advisor/Consultant Neelima Jain, PhD, GSK: Employee|GSK: Stocks/Bonds (Public Company) Heidi Swygard, MD, GSK: Stocks/Bonds (Public Company)|ViiV Healthcare: Employee Todd McKeon, PharmD, GSK: Stocks/Bonds (Public Company)|ViiV Healthcare: Employee Annemiek de Ruiter, MBBS FRCP, GSK: Stocks/Bonds (Public Company)|ViiV Healthcare: Employee Maggie Czarnogorski, MD MPH, GSK: Stocks/Bonds (Public Company)|ViiV Healthcare: Employee Nanlesta Pilgrim, PhD, GSK: Stocks/Bonds (Public Company)|ViiV Healthcare: Employee

